# Improving self-referral for diabetes care following hypoglycaemic emergencies: a feasibility study with linked patient data analysis

**DOI:** 10.1186/s12873-016-0078-1

**Published:** 2016-02-18

**Authors:** Edward A. S. Duncan, David Fitzpatrick

**Affiliations:** Nursing, Midwifery & Allied Health Professions Research Unit, Scion House, University of Stirling FK9 4NF, Scotland, UK; Clinical Research Paramedic (Scottish Ambulance Service), Nursing, Midwifery & Allied Health Professions Research Unit, Scion House, University of Stirling FK9 4NF, Scotland, UK

**Keywords:** Hypoglycaemia, Pre-hospital, Prehospital, Ambulance, Paramedic, Data collection/methods, Telehealth, Linked data

## Abstract

**Background:**

Hypoglycaemia is a common and potentially life threatening consequence of insulin and sulphonylurea treated Diabetes. Some severe hypoglycaemic events result in emergency ambulance attendance. Many of these patients are treated at home and do not require immediate transportation to an Emergency Department. However only 27-37 % of patients then follow up their care with a diabetes specialist. Consequently repeat severe hypoglycaemic events occur.

**Methods:**

The intervention was implemented for 8 months, using a prospective cohort design with a historic control, in one Scottish Health Board in 2012. Data was collected using postal survey questionnaires to patients and ambulance clinicians, telephone survey follow-up questions to patients. Scottish Ambulance Service electronic records were linked with the SCI-Diabetes database of patient records to enable objective measurement of follow-up behaviour.

**Results:**

Ambulance clinicians’ (*n* = 92) awareness of the intervention was high and both the prompt card and telephone call components of the intervention were delivered to most eligible patients. The intervention was perceived as highly acceptable to patients (*n* = 37), and very useful by both patients and ambulance clinicians. However, comparison of patient follow-up behaviours using linked-data (*n* = 205), suggest that the intervention was unsuccessful in improving rates of patients’ following up their care.

**Conclusions:**

This study shows that the intervention is implementable, highly acceptable to patients, and considered very useful by both patients and ambulance clinicians. However, preliminary evidence of effectiveness is not encouraging. The study’s novel use of linking existing clinical data for outcome measurement exposed challenges in the feasibility of using this data for intervention development and evaluation. Future research should examine challenges to the successful testing and effectiveness of the intervention. Revisions are likely to be required, both to study design and the optimisation of the intervention’s content and components.

**Electronic supplementary material:**

The online version of this article (doi:10.1186/s12873-016-0078-1) contains supplementary material, which is available to authorized users.

## Background

Hypoglycaemia is a common adverse effect of insulin and sulphonylurea treated Diabetes Mellitus. It can be experienced to varying degrees. Mild to moderate hypoglycaemia can be self-treated. Severe hypoglycaemia, defined as a person requiring external assistance to treat, is a serious condition which can lead to coma, seizure, and death. The annual prevalence of severe hypoglycaemia among patients with type 1 diabetes mellitus is approximately 30 %, with the incidence increasing due to disease duration, amongst other factors. In type 2 diabetes mellitus the prevalence is less, but incidence similarly rises with length of duration of insulin or sulphonylurea therapy [1]. Recurrent episodes of severe hypoglycaemia can cause permanent cognitive impairment leading to cognitive decline and an acceleration of the onset of dementia [1].

Though the majority of severe hypoglycaemic emergencies are treated by family and friends, some result in an emergency ambulance attendance. Severe hypoglycaemic events in people with Diabetes account for 0.5 %–1.02 % of all emergency ambulance call outs in the United Kingdom per annum [2–5]. Whilst this percentage is small, the denominator of emergency ambulance callouts across the United Kingdom is substantial: There are approximately 9.08 million emergency ambulance callouts in England and 700,000 emergency callouts in Scotland each year [6, 7]. This means that there are between 48,400 and 98,736 severe hypoglycaemic emergencies attended by ambulance clinicians each year, across England and Scotland alone. The vast majority (63 %–73 %) of these patients are not transported to hospital [2, 4, 5].

Many people (63 %–73 %) remain at home following ambulance clinician treatment for a severe hypoglycaemic event [3–5]. Though immediate attendance at an emergency department is unnecessary, it is still advisable for these people to follow-up their care with an appropriate diabetes specialist. Without doing so they are at risk of further hypoglycaemic emergencies. Internationally, studies have shown that 2-7 % of these patients require repeat ambulance attendance due to a recurrent hypoglycaemic emergency episode within two days [3–5, 8–11]. There is growing evidence that this proportion increases over time. A recent retrospective review of the incidence of hypoglycaemic events requiring an emergency ambulance, found that 7 % had repeat hypoglycaemic event within 7 days, and 11 % had a repeat hypoglycaemic event within 14 days [12].

Specialist diabetes follow-up subsequent to a severe hypoglycaemic emergency is likely to improve patients’ understanding of the cause of their severe hypoglycaemic episode, avoid repeat hypoglycaemic events, and receive advice on a course of action to improve diabetes self-management. A cross sectional questionnaire survey of 128 patients who had been attended by the ambulance service due to a hypoglycaemic emergency identified that only 37 % of patients later contacted their primary care service [13]. A systematic review of pre-hospital post-hypoglycaemic emergency patient care recommended that evidence-based interventions to increase patient rates of follow-up be developed and evaluated [14].

There are many reasons why people may not follow-up their care. People may not understand or remember the advice they are given by ambulance clinicians due to post-hypoglycaemic cognitive dysfunction. People may also underestimate the importance of attending their diabetic care provider, or feel embarrassed to do so in the wake of a hypoglycaemic emergency, perceiving it as some sort of failure [13]. One potentially effective, and inexpensive, intervention that could address these issues is the introduction of written prompts for patients and a follow-up telephone call to check on recovery and reinforce advice to attend their regular diabetes care provider for review. Written and telephone prompts are recognised as effective behaviour change mechanisms to address individuals action planning, such as attendance at the appropriate diabetic care provider following a hypoglycaemic emergency [15].

This study reports on the feasibility and initial evaluation of an intervention comprising written and telephone prompts designed to improve patient rates of attendance for post hypoglycaemic emergency follow-up care with their diabetes care provider. We aimed to assess a) the feasibility and acceptability of implementing the intervention to patients and ambulance clinicians; and b) the feasibility of collecting outcome data (attendance for follow-up) using linked data analysis.

## Methods

### Design

The study design was informed by the MRC Framework for Complex Interventions [16]. A prospective cohort design with a historic control was used. This enabled us to investigate the intervention’s feasibility, acceptability, and implementation. Data were collected using postal survey questionnaires to patients and ambulance clinicians, telephone survey follow-up questions to patients, and linked-data analysis. Ethical approval for the study was obtained from the West of Scotland Research Ethics Service (REC reference: 11/WS/0072).

### Participants

Eligible patients were adults (>18 years of age) attended by the ambulance service due to a hypoglycaemic emergency. All ambulance clinicians (i.e. paramedics and ambulance technicians) who worked within the study setting during the intervention period were eligible and invited to complete the study feasibility questionnaire.

### Setting

The study took place in one regional health board area of Scotland. The intervention was delivered by ambulance clinicians over an eight month period, from January 2012 to August 2012.

### Intervention materials

The intervention’s design was informed by 26 qualitative interviews with adults (15 male, 11 female) who had recently experienced a hypoglycaemic emergency and had been attended by ambulance clinicians. Data was transcribed verbatim and analysed using Framework Analysis [17]. This provided an in-depth understanding of reasons people do not follow-up their care [12].

The intervention had two components: a prompt card and a follow-up telephone call. The prompt-card was developed during a one day facilitated workshop attended by people with diabetes (*n* = 4), ambulance clinicians and managers (*n* = 11), diabetic specialist nurses (*n* = 3), study steering group members (*n* = 7), and general practitioners (*n* = 2). A co-design and storytelling process [18], informed by the qualitative interviews, was used to generate the content and design of a prompt card that would be left with patients by ambulance clinicians following attendance for a hypoglycaemic emergency. Attention was paid to ensuring that the design of the card would ease its use by ambulance clinicians, who already have to carry substantial amounts of equipment to emergency calls. The card highlighted the benefits of patients’ following-up their care. Drafts of the prompt card intervention were piloted following the workshop with five people who had experienced hypoglycaemic emergencies, before being finalised. This piloting process led to the inclusion of an integrated peel off sticker, that was removed from the prompt card by the ambulance clinician and left in a prominent position (e.g. a fridge door or next to the telephone) to act as an additional prompt (See Additional file 1).

The follow-up telephone call aimed to provide further reinforcement of the importance of patients’ following-up their care, and to reassure ambulance clinicians that they would not be the final point of contact. Following on from the workshop a series of meetings were conducted with staff from NHS24 (Scotland’s national telehealth and telecare organisation) who would make the calls, on behalf of the ambulance service. These meetings used both the qualitative interview findings, and the workshop output as the basis for developing a structured script to be used by call operators when phoning patients.

### Intervention procedures

Every ambulance station in the study area (*n* = 7) was visited by a research paramedic (DF) prior to the intervention’s commencement. The purpose of these visits was to deliver study materials, inform ambulance clinicians about the study, and provided them with information of how and when to provide the study prompt card. Each ambulance clinician was provided with a credit card sized study aide memoir to remind them of key study information. Ambulance clinicians were instructed to give the prompt card, to all adult patients who they attended due to a hypoglycaemic emergency and had left at home, at the end of their visit. As part of this process, ambulance clinicians were asked to leave the study sticker in a prominent location in each patient’s house. Patients were not required to consent to receive this information, as it was considered to be an enhancement of current practice. Consent for use of sticker in patients houses was sought before use on each occasion. Details of each ambulance service attendance due to a hypoglycaemic emergency were, with the patient’s consent, then passed to NHS24. Follow-up phone calls were delivered by non-clinical NHS24 call handlers. Patients were called approximately 72 h after their hypoglycaemic emergency. If necessary, up to three attempts were made to contact each patient.

### Data collection tools

Patients’ perspectives on the feasibility and acceptability of the intervention were collected through a postal questionnaire and telephone contact by NHS24. The questionnaire covered three topic areas: general information received after the emergency event; specific questions about the prompt card and follow-up telephone call comprehensiveness and ease of use; and follow-up care they sought or received. The questionnaire was piloted with a group of people (*n* = 4) who had previously experienced a hypoglycaemic emergency. The pilot group were asked to comment on the overall style and appeal of the questionnaire, the relevance and clarity of each question, and the time taken to complete. Feedback led to minor changes to wording and presentation. Questionnaires and response paid envelopes were sent within 1 week of the hypoglycaemic event to all patients who had experienced a hypoglycaemic emergency and were not transported to hospital. A reminder was sent following 7 days. Consent to participate was implied through the return of a completed questionnaire. Rates of telephone contact with patients and whether participants they spoke to reported receiving the prompt card were recorded by NHS 24 staff on an Excel spreadsheet.

A questionnaire was also designed to measure ambulance clinicians’ perspectives on the feasibility and acceptability of the intervention. The questionnaire covered three topic areas: awareness of the intervention; fidelity of intervention delivery; and perceived usefulness of the intervention. The questionnaire was piloted with a group of ambulance clinicians from differing geographical areas (*n* = 5) to the study intervention setting. Feedback led to minor changes to wording. The questionnaire was sent to all ambulance clinicians within the study catchment area (*n* = 206) at the conclusion of the intervention period. Reminders were sent to all participants after 7 days. Consent to participate was implied through the return of a completed questionnaire.

We conducted a record linkage study of diabetes related emergency call patient data (Advanced Medical Dispatch Code 13, Scottish Ambulance Service) with The Scottish Care Information – Diabetes Collaboration (SCI-Diabetes) database. Pre-hospital studies have demonstrated that between 42 % to 98 % of ambulance calls for diabetes related emergencies are for hypoglycaemia [3, 8, 10]. A random sample of 50 diabetes related emergency calls were cross-referenced with ambulance clinicians patient report forms. This determined that almost all randomly selected diabetes related calls were diagnostic of hypoglycaemia. SCI-Diabetes provides a fully integrated shared electronic patient record (including Primary Care) to support treatment of NHSScotland patients with Diabetes. Data were linked and anonymised by the University of Dundee Health informatics Centre (HIC). Data were linked from two time periods: July 2010 - December 2011; and January 2012 August 2012 inclusive. Annonymised data were securely stored on the HIC Safe Haven which was remotely accessed for analysis. The first time period acted as a control for the second when the intervention was delivered. The primary outcome we wished to measure was “attendance” for diabetes follow-up after being attended by ambulance clinicians for a severe hypoglycaemic event. SCI-Diabetes does not have a variable for ‘attendance’. Following discussion with consultant diabetologists familiar with SCI-Diabetes, blood pressure records were combined with HbA1c records and the date of either of these events was used a proxy variable for engagement with diabetes care.

### Data analysis

Questionnaire and NHS24 Excel data were entered into the Statistical Package for the Social Sciences (SPSS) (v.21). Descriptive statistics were used to analyse the results of the questionnaire and telephone contact data. Chi Square analysis was used to compare the proportions of patients who received a prompt card with those who reported following up their care with their diabetes care provider.

Patient level linked-data were imported into R, a software environment for statistical computing and graphics, and the psudonomised patient ID and date of event were extracted. For each patient the time between the ambulance visit (trigger event) and a proxy event was calculated, these data were then used to compare the number of follow-up attendances post trigger event for the control and intervention groups. This analysis was carried out for follow-up attendances occurring 14, 21 and 28 days after the trigger events.

## Results

During the intervention period, the Scottish Ambulance Service received 280 calls for hypoglycaemia related emergencies within the study catchment area. Of the people who were treated 107 (38 %) were not transported to the Emergency Department and were included in analysis.

In keeping with findings from related studies [2, 3, 19] more males than females required emergency assistance (156; 58.3 % vs. 112; 39.6 %). Mean age of patients was 55 (SD 22.15). There was no significant difference in mean age between male and female participants (56.5 vs 54.0, *x*^2^ = .869).

### Feasibility and acceptability of implementing the intervention

#### Patient participants

Thirty-seven participants (34 %) returned completed questionnaires. Thirty-four (92 %) reported receiving at least one part of the intervention: 25 (68 %) reported receiving the prompt card and 23 (64 %) the follow-up telephone call. Fifteen participants (40 %) reported receiving both parts. Only three (8 %), reported receiving neither the card nor phone call. Only one participant stated they had opted out of receiving the follow-up call.

All participants who received the prompt card stated they found it either ‘easy to understand’, ‘very easy to understand’ or ‘extremely easy to understand’ (29.7 %, *n* = 11; 21.6 %, *n* = 8; 16.2 %, *n* = 6). Participants’ were positive about the intervention, with most suggesting it was useful to extremely useful. Responses regarding the telephone call component were more varied (Table 1).

**Table 1:** Ease of use of intervention by patient participants

	Total responses	Not at all useful	Only moderately useful	Useful	Very useful	Extremely useful
Card	19	1 (3 %)	-	7 (19 %)	5 (14 %)	6 (16 %)
Sticker	24	1 (3 %)	-	11 (30 %)	6 (16 %)	6 (16 %)
Phone call	27	5 (18 %)	6 (22 %)	9 (33 %)	5 (18 %)	2 (7 %)

Follow-up telephone contact was achieved by NHS24 staff with 71/107 (69 %) of patients (missing data 4 (4 %). The majority of patients were contacted on the first attempt (42/107, 40 %), 13 (12 %) were contacted on the second attempt, and 16 (15 %) were contacted on the third attempt. Most participants (22/30, 73 %) reported that the most appropriate timing of the follow-up telephone call was 1–3 days post hypoglycaemic event. Three (10 %) suggested the follow-up telephone call should be at day one, and 5 (17 %) suggested between 3–7 days. Patient participants were asked by the call handler if they had already followed up their care with their diabetes care provider (i.e. following receipt of the prompt card alone). More patients reported following up their care if they had received the prompt card (28/36, 78 %) than if they had not (13/23 (57 %), but this was not significant (*χ*^2^(*df* =1, *n* = 59) = 2.99; *p* = .084).

Thirty-one participants responded to the questionnaire regarding follow-up care with diabetes services. Nineteen (61 %) stated that they had followed up care, 12 (39 %) stated that they had not. Most (*n* = 15, 78 %) felt it was ‘easy’, ‘very easy’ or ‘extremely easy’ to make a follow-up appointment with 4 (21 %) suggesting it was ‘not at all easy’ and 1 (5 %) finding it only ‘moderately easy’.

#### Ambulance clinicians

Ninety-two (44 %) ambulance clinicians returned a completed questionnaire. Eighty-four (91 %) had attended a hypoglycaemic emergency during the study period, six (7 %) had not and two (2 %) could not remember. All participants (100 %) stated they were aware of the intervention.

When asked whether they left the prompt card with the patients who were not transported to hospital 91 (99 %) responded. Sixty three (69 %) stated they ‘always’ left the prompt card, 12 (13 %) said ‘usually’, 9 (10 %) ‘sometimes’ and two (2 %) ‘never’. We wanted to find out if ambulance clinicians used the sticker. Almost all participants (91 %, *n* = 84) answered this question. Fifty-two (57 %) said they used it ‘always’ or ‘usually’ (*n* = 43, 46 %; *n* = 9, 10 %,) and 32 (38 %) suggested they used it ‘sometimes’ or ‘never’ (*n* = 15,16 %; *n* = 17,19 %).

Ambulance clinicians were asked how useful they felt each element (i.e. the Prompt Card; Sticker; and Follow-up telephone call) of the intervention to be. Usefulness was measured on a 5 point Likert scale with ‘1’ labelled as ‘not at all useful’ and 5 labelled as ‘extremely useful’. The prompt card was perceived to be the most useful (Mean 4.03, SD 1.32), followed by the follow-up telephone call (Mean 3.91, SD 1.44), and the sticker (Mean 3.54, SD 1.51).

#### Data linkage

Two hundred and five of the 280 cases were data linked. Seventy-five missing cases were unable to be probabilistically matched. The lack of an “attendance” field made measurement of our primary outcome challenging. Proxy measures of blood pressure and HBA1c were required to be used. This meant that individuals who attended a clinic, or phoned for advice, and received only verbal advice were not counted as having “attended” in our analysis.

Comparisons of diabetes care provider attendance before and during the intervention suggest that the intervention resulted in no increase in diabetes follow-up at 14, 21 or 28 days following their hypoglycaemic emergency (Table 2).

**Table 2:** Comparison of follow-up appointment made before and during the intervention period

	14 days post trigger event			21 days post trigger event			28 days post trigger event		
Follow-up	Control	Intervention	Total	Control	Intervention	Total	Control	Intervention	Total
No visit	255 (83 %)	172 (84 %)	427	231 (76 %)	164 (80 %)	395	217 (71 %)	158 (77 %)	375
Visit	51 (17 %)	33 (16 %)	84	75 (24 %)	41 (20 %)	116	89 (29 %)	47 (23 %)	136
Total	306 (100 %)	205 (100 %)	511	306 (100 %)	205 (100 %)	511	306 (100 %)	205 (100 %)	511
	*χ* ^2^(*df* = 1, *n* = 511) = 0.002; *p* = .961; ϕ = .002 OR = 0.959 (95 % CI 0.594 – 1.548)			*χ* ^2^(*df* = 1, *n* = 511) = 1.177; *p* = .228; ϕ = .048 OR = 0.770 (95 % CI 0.501 – 1.184)			*χ* ^2^(*df* = 1, *n* = 511) = 2.079; *p* = .149; ϕ = .064 OR = 0.725 (95 % CI 0.482 – 1.091)		

### The advantages and disadvantages of the intervention

The advantages and disadvantages of the intervention are summarised, as a balance sheet, in Table 3. This highlights issues that are of central importance and which will assist in our future trial design and research.

**Table 3:** Balance sheet of quantitative and qualitative advantages and disadvantages of the duel intervention to improve self-referral following hypoglycaemic emergency

Advantages	Disadvantages	Comments
Patients’ experience		
Prompt card		
The prompt card was perceived to be highly acceptable, and respondents indicated that it had prompted them follow-up their care:- ➢ 67 % said that they were left the prompt card. ➢ 63 % found the card to be ‘useful to extremely useful’. ➢ 96 % who received the prompt card said it was ‘useful to extremely useful’. ➢ 38 % who were left the prompt card, said that it encouraged them to follow-up their care. ➢ 42 % who were left the prompt card said that the verbal advice encouraged their follow-up care. ➢ There was a 2–3 fold increase in self-reported self-referral to diabetic care providers following receipt of prompt card.	➢ The ‘full’ intervention (prompt card + NHS24 phone call) was reported as being delivered 36 % of the time. ➢ There was no evidence from linked data analysis, or participant reports, that the intervention had any significant effect on increasing the proportion of patients who attend their usual diabetes care provider for follow up for a review of their medication and other risk factors following a hypoglycaemic emergency attended by the ambulance service.	
Telephone call		
NHS24 attempted to contact all patients. ➢ 75 % of all calls made were answered. ➢ 40 % of patients were contacted on the first call ➢ 69 % of answered calls were directly with the patient, 13 % were with close relatives. ➢ 67 % of respondents found the follow-up phone-call “useful- extremely useful”. ➢ 63 % of individuals found the card to be ‘useful to extremely useful’.	➢ Of the calls that were answered 12 % of answered calls were third party individuals’ with no knowledge of the patient. ➢ A few participants (n = 6) stated they would not follow-up their care when contacted by NHS24.	Third party call recipients are likely to have been at locations that were not a patient’s home (e.g. a supermarket). The ambulance service records the telephone number of the call, not patients’ home or mobile number. Despite very positive comments about the card many did not appear to follow-up up their care.
Ambulance clinicians experience (44 % [n = 92) completed the questionnaire, 91 % had attended a hypoglycaemic emergency during the study period).		
Pros	Cons	Comments
➢ All respondents rated the prompt card & telephone follow- up ‘very - highly useful’ ➢ 83 % felt the location of the prompt card in the response bag made them easier to access and 77 % felt this increased the likelihood that they would pass the prompt card onto patients. ➢ 69 % said they always left the prompt card. 13 % said the usually left the prompt card ➢ 62 % suggested they always or usually left the prompt card sticker. ➢ 96 % were aware that the patient would receive a follow- up telephone call. ➢ 73 % said they always told the patient to expect a follow- up telephone call.	➢ Prompt cards were reported as declined by patients or carers on 4 occasions. ➢ Prompt cards were forgotten to be left on 8 occasions ➢ Prompt cards were not available on 6 occasions. ➢ 38 % said they ‘sometimes’ or ‘never’ used it ➢ 11 % said they ‘sometimes’ or ‘never’ told the patient to expect a follow-up telephone call.	The intervention was generally perceived to be very acceptable and feasible in practice.

## Discussion

The aim of this study was to investigate the feasibility and conduct a preliminary evaluation of an intervention comprising written and telephone prompts designed to improve patient rates of attendance for post hypoglycaemic emergency follow-up care with their diabetes care provider. Our findings suggest that the intervention was generally well implemented. Ambulance clinicians’ awareness of the intervention was high and both the prompt card and telephone call components of the intervention were delivered to most eligible patients. Overall, the intervention was perceived as highly acceptable to patients and viewed as very useful by both patients and ambulance clinicians. The novel mechanism of pre-hospital care intervention delivery -a research based prompt card combined with a follow-up telephone call - can therefore be viewed as a success. Use of telephone follow-up, in particular, may be a valuable mechanism by which to lessen ambulance clinicians’ fears of leaving patients at home, a known barrier to alternative pre-hospital care pathway implementation [20].

The preliminary evaluation of the intervention’s potential effectiveness indicates that it may make no difference to patient follow-up behaviour. This study was not designed to definitively answer the question of the intervention’s effectiveness. However, the cross sectional comparison of patient follow-up behaviours using linked data and patient responses to receiving the intervention, suggest that the intervention was unsuccessful in improving rates of patients’ following up their care. This is a potentially significant problem. Close consideration of both study method and findings are required to guide decision making about study revisions before decisions are made whether or not to take the intervention forward in an effectiveness trial. Detailed guidance to support such decision making has been lacking. However, a process to support decision making following feasibility and piloting stages of an intervention, known as AdePT, has recently been developed [21]. The ADePT process suggests that there are three core problem types that need to be considered: issues that are only a problem for the trial; issues that are likely to be a problem for both the trial and the real world; and issues that are only likely to be a problem for the real world [22]. Our study findings suggest that there are problems that relate to the trial alone, and which are both real world and trial related.

The use of linked-data as a means of primary outcome measurement is a trial related problem. SCI-Diabetes is a rich source of clinical diabetes data. It gathers real time clinical information about almost all diagnosed cases of diabetes in Scotland, UK, and has been successfully mined for a range of large epidemiological studies [22–24]. To our knowledge, however, this is the first occasion that the SCI Diabetes dataset has been used as a means of primary outcome evaluation in an intervention development study. Attendance for follow-up care was the primary outcome we wished to measure. The lack of an ‘attendance’ field within the dataset proved problematic. Proxy measures of blood pressure and HBA1c records were used, as these were believed to be the most likely fields to be completed when a patient followed up their care. However it is possible that patient attendances were missed as these measures may not always have been taken. Furthermore, SCI-Diabetes has no means of recording patients telephoning for advice and it is possible that some patients may have phoned following their hypoglycaemic emergency, rather than attending in person. Nineteen (61 %) of the intervention participants reported following up their care after their hypoglycaemic emergency. This figure appears at odds with the SCI-Diabetes evaluation, however it is impossible to know whether this self-reported figure was missed by our proxy measure assessment, or whether it reflects a social desirability response on behalf of the participants. These issues make the use of SCI-Diabetes as the means of primary outcome measurement in a future trial of this intervention problematic. An assessment of alternative options is required. This should take the potential effectiveness and feasibility of primary outcome measurement into consideration before a decision is made.

The content of the intervention as a mechanism to change patients’ follow-up behaviour is a theoretical and trial related problem. Whilst the mechanism of the prompt card and follow-up telephone call appears to be viewed as acceptable and useful by patients and ambulance clinicians alike, it does not appear to have significantly changed patients’ follow-up behaviour. This may be because appropriate behavioural change theories were not fully optimised within the content of the intervention. Behavioural change theories [25, 26] were closely considered in relation to the mechanisms of action relating to the form of the prompt card and telephone call components of the intervention. However, less importance was placed on the use of behavioural change theories in guiding the content of both components. Enhancing the use of established behaviour change theories in the content of the intervention may prove more successful in changing patients’ follow-up behaviour. Our intervention relies on patients’ changing their follow-up care behaviours. An alternative intervention approach is to empower clinicians with information about patients’ hypoglycaemic emergencies and undertake a diabetes service led follow-up intervention [27]. An evaluation of such an intervention is currently underway [28]. Diabetes service led care pathway interventions, however, will still require patients to understand the importance of the intervention they are being offered and be sufficiently motivated to attend for a follow-up appointment. Ultimately, therefore, both patient and service led intervention approaches may be required to change patients’ follow-up behaviour. Further consideration is therefore required to optimise the intervention components and content for delivery in a future trial.

### Strengths and limitations

This study has demonstrated that the dual intervention of prompt card delivery by ambulance clinicians and telephone call follow-up can be successfully implemented, is highly acceptable and perceived as useful in pre-hospital emergency care. Use of SCI-Diabetes was a novel approach to outcome measurement and key lessons have been learnt through this study that highlight the limitations of using this type of data for outcome measurement in a patient and clinician level intervention. Use of blood pressure and HBA1C variables as proxy measures for patient attendance may have led to a misclassification bias as cases could have been missed where either patients attended but these measures were not taken, or patients telephoned their diabetes specialist for advice, in which case these variables could not have been provided.

Despite the use of a range of recognised techniques (e.g. inclusion of pens, response paid envelopes and reminders) to improve response rates [29, 30], the low percentage of questionnaire returns from patients (*n* = 37, 34 %) and ambulance clinicians (*n* = 92, 44 %) limit the transferability of the findings to the wider population.

## Conclusions

This dual patient prompt card and tele-health intervention to improve rates of follow-up care following a hypoglycaemic emergency that has been attended by ambulance clinicians contained two very original components: the use of the telehealth organisation NHS24 to proactively telephone patients following ambulance service attendance, and the use of SCI-Diabetes as a potential means of objective measurement of the primary outcome data.

The intervention was implementable, highly acceptable to patients, and considered useful to very useful by the majority of patients and ambulance clinicians. Proactive telephone follow-up by NHS24 was feasible and perceived as useful by patients. The study’s novel use of linking existing clinical data for use in outcome measurement exposed challenges of using this data in intervention development and evaluation studies. Future research should examine trial, theoretical, and pragmatic related challenges to the successful testing and effectiveness of the intervention. Revisions are required both to study design, in particular the method by which primary outcome measurement is gathered, and to the optimisation of the intervention’s content and components.

## Additional file

Additional file 1: Intervention Prompt Card. (PDF 118 kb)
