# Supplementary material for: Improving self-referral for diabetes care following hypoglycaemic emergencies: a feasibility study with linked patient data analysis
Source: BMC Emerg Med. 2016 Feb 18;16:13. doi: 10.1186/s12873-016-0078-1 (PMC4757997; doi:10.1186/s12873-016-0078-1)
Supplement: Additional file 1: — Intervention Prompt Card. (PDF 118 kb) [file 12873_2016_78_MOESM1_ESM.pdf]

You have required emergency assistance for a severe hypoglycaemic event

It is very important that you now inform the person who provides your diabetes care about this event. The ambulance service will not inform them that they have been to see you.

Following up your diabetes care is very important as you may be at risk of experiencing another hypo in the next few days or weeks. Seeing a relevant healthcare professional can:

- help determine the cause of your hypo
- lead to a review of your current medications and potentially make some helpful changes
- provide personalised expert advice on hypo prevention

Although the ambulance has left, you are not alone!

In about three days you will receive a telephone call from the ambulance service.

This is to see how you are and to encourage you to follow up your care.

If you **would not** like them to contact you, please call this free-phone number and leave a message:

☎ **0800 8620 0234**

For advice about Diabetes

If you would like further information about Diabetes, you may find it useful to contact Diabetes UK Scotland:

Careline Scotland:
☎ **0845 120 2960**
www.diabetes.org.uk

Diabetes UK Scotland

Details of your treatment:

Date:		Arrival time:
**BM test results on:**		
arrival:		
recovery:		
**Treatment given:**		
Glucagon		1mg
10% I.V. Glucose		mg
Glucogel		tube(s)

IMPORTANT:
You have had a severe Hypo!

Tell your diabetes care provider about this event as soon as you can

If you become seriously ill again **dial 999** and ask for an ambulance.
